# Experimental Study on Fatigue Behaviour of Shot-Peened Open-Hole Steel Plates

**DOI:** 10.3390/ma10090996

**Published:** 2017-08-25

**Authors:** Zhi-Yu Wang, Qing-Yuan Wang, Mengqin Cao

**Affiliations:** 1 Department of Civil Engineering, Sichuan University, Chengdu 610065, China; zywang@scu.edu.cn (Z.-Y.W.); 18224482076@139.com (M.C.); 2 Sichuan Provincial Key Laboratory of Failure Mechanics and Engineering Disaster Prevention & Mitigation, Sichuan University, Chengdu 610065, China; 3 School of Architecture and Civil Engineering, Chengdu University, Chengdu 610106, China

**Keywords:** open-hole, shot peening, fatigue behaviour, fracture mechanics

## Abstract

This paper presents an experimental study on the fatigue behaviour of shot-peened open-hole plates with Q345 steel. The beneficial effects induced by shot peening on the fatigue life improvement are highlighted. The characteristic fatigue crack initiation and propagation modes of open-hole details under fatigue loading are revealed. The surface hardening effect brought by the shot peening is analyzed from the aspects of in-depth micro-hardness and compressive residual stress. The fatigue life results are evaluated and related design suggestions are made as a comparison with codified detail categories. In particular, a fracture mechanics theory-based method is proposed and demonstrated its validity in predicting the fatigue life of studied shot-peened open-hole details.

## 1. Introduction

Bolted connections in steel bridges are subjected to fatigue loading due to continuous vehicular traffic. In most cases, cracks are prone to take place in the vicinity of the bolt hole where there is a geometric discontinuity. If the crack continues to propagate and is left unmaintained, catastrophic failure may occur. Regarding this, several methods in retrofit and improvement of the behaviour of the open-hole details have been highlighted in recent research works [1,2]. Obviously, the effectiveness of the control over the crack initiation is important for the fatigue life improvement of these structural components.

Shot peening has been extensively applied with the purpose of fatigue life improvement since the work-hardening and considerable compressive residual stresses can be introduced as a result of near-surface plastic deformation [3,4]. The effect of surface treatment on the fatigue behaviour of steel components has been highlighted by most recent studies. Fernandez-Pariente et al. [5] experimentally investigated the fatigue behaviour of nitrided and shot-peened steel with artificial small surface defects. It was shown that the shot peening is able to increase the fatigue strength of nitride specimens with pre-existing defects regardless of the defect size and generation method. Also, the fatigue strength of the specimens with larger surface defects obtained by electro-erosion is bigger than the one of specimens with smaller defects obtained by indentation. Later, the effect of combining severe shot peening and nitriding on the fatigue limit of low-alloy steel was observed by Hassani-Gangaraj et al. [6] to be useful for the improvement of hardening, residual stress and nitrogen diffusion as compared with single treatment but uncertain in contrast to nitride smooth specimens. Subsequently, Sakamoto et al. [7] observed that the fatigue fracture of test specimens was caused by crack propagations which in turn affected the fatigue limit of shot peening specimens. Roughness, hardness and residualstress analysis were also used by Závodská et al. [8] for characterizing the strongly deformed surface layer. The reported test results showed increased fatigue strength and limit in the high cycle region with the redistribution of residual stresses. Recently, the surface integrity was experimentally confirmed by Gerin et al. [9] as a major impact on fatigue behaviour. The forging defects are detrimental in fatigue and lower the fatigue strength with larger defects having a greater impact. Purohita et al. [10] performed finite element modelling for shot peening specimens, varying shot speed, shot size, as well as the angle of impact. It was found that the reduction of the last measure leads to ineffective benefit of shot peening, while the increase of the others significantly enlarges the effect of residual stresses.

To assess the compressive residual stresses induced by shot peening, Zhuang and Halford [11] developed a Bauschinger effect-based relaxation model which showed a good comparison with numerical simulation during the early cyclic loading stage of low cyclic fatigue. Liu and Pang [12] investigated the shot-peened material characterized by multiaxial stress state in which out-of-plane stress is dominant compared with in-plane stress. Only the effect of the residual stress was considered in their suggested model. Additionally, the fatigue behaviour reliability of shot-peened metallic parts through a probabilistic approach was recently studied by Seddik et al. [13]. In the application of the theoretical model, the elastic concentration factor was studied by Li et al. [14] and Rodopoulos et al. [15]. The crack growth related to an equivalent initial flaw was studied by Xiang et al. [16]. Also, this method was proved by Liu and Mahadevan [17] as efficient in combination with probabilistic crack growth analysis to predict the fatigue life of smooth specimens. In addition, conventional methods for fatigue crack growth as reported by Refs. [18,19,20,21,22,23] have also been adopted in the study of fatigue behaviour of open-hole plates.

Despite significant research of shot peening action on the stress characteristics of unnotched specimens, however, its resultant effect on the open-hole details has not been clearly explained. The fatigue failure of open-hole has been highlighted by most researchers. Recho and Brozzetti [18] studied the laws of fatigue damage and variable cyclic stresses-induced cumulative damage laws which can be used for open-hole steel plates. Later, this method has been adopted for fatigue-induced failure of similar plate mounted on a harbour crane. Recently, the authors have also conducted a series of fatigue experimental research and comprehensive review of research work can be referred to [1,2,24]. For the improvement of fatigue strength, some other techniques, such as bolt clamp-up and carbon fibre-reinforced polymer (CFRP) strengthening, have also been recently highlighted by the authors [24] and other researchers. In [24], the combination of bolt clamp-up action was considered in the improvement of fatigue strength. For the test specimen with centre hole covered with CFRP as reported in [25], it was shown an increased fatigue life of 54% over the un-retrofitted ones, while that only applied on either side of the crack starter exhibited only 25% life enhancement. It is noted that the fatigue loading can reduce the bond strength between normal modulus CFRP and steel by approximately 20% to 30%, whereas such a reduction becomes insignificant in the case of high modulus CFRP [26]. Regarding this, it is important to understand the advantage of shot peening over some other techniques in the increase of fatigue life of open-hole steel plates.

In this paper, the fatigue behaviour of open-hole steel plates induced by shot peening is studied. The aspects of failure mode will be characterized at crack initiation sites with scanning electron microscopy (SEM), and then, the fatigue life of the shot-peened specimens (SP) is compared with as-machined (AM) or non-peened specimens. Theoretical equations are developed in the evaluation of the improvement of fatigue life results of shot-peened open-hole details. The main concerns are given herein not only for the fatigue failure behaviour, but also for the theoretical method in the evaluation of fatigue strength of open-hole details which differentiates this presented research work from the others reported in the literature.

## 2. Material and Experimental Procedure

Material was received in the form of a 6.0 mm thick plate which conforms to Grade 345B low alloy steel in the Chinese national standard GB/T1591. The chemical composition and mechanical property are listed in Table 1. For comparison, the steel material of S460M in [27] is also given herein for reference and discussion in the subsequent section.

**Figure 1:**
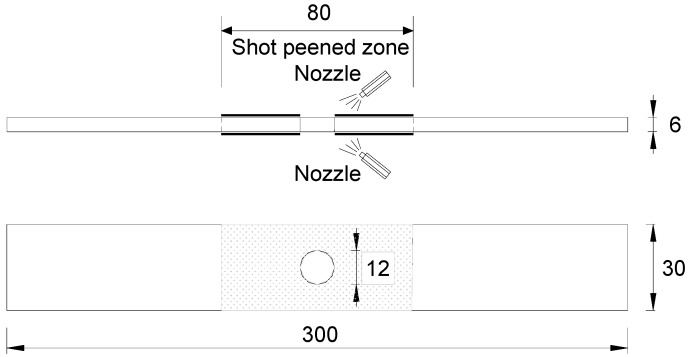
Dimension and design of test specimen (unit: mm).

To avoid contamination of the test specimens from loose or broken inclusions, porosity, clusters, etc. due to hot rolling, a 10 mm layer was cut and removed from each side of the plate before machining. The dimension of open-hole details is shown in Figure 1. The 6 mm thick steel plate was machined to its design dimension of 300 mm long and 30 mm wide. The bolt clearance holes of 12 mm diameter were cut at the centre of the plate using a standard laser cutter. The laser cutting process is started by completely penetrating the metal workpiece, and then the laser beam moves around, melting the materials as it passes. Meanwhile, a stream of gas blows the melted materials downwards out of cut to form a hole. In contrast, the punched hole is fabricated by a shearing operation as a male punch is forced through the work piece and through a female die. Related parameters are the size of the hole to be punched and the thickness and shear strength of the base steel. All tests were performed using specimens oriented with the tension axis parallel to the rolling direction and the test specimen surface normal to the rolling plane.

The idea here is that by retarding the crack at the edge of the open-hole could be expected by means of shot-peening. Prior to shot peening, the surface condition was determined by roughness & micro-hardness measurement using profiled geometry. During shot peening process, double sides of the steel plate near the bolt clearance hole, as shown in Figure 1, was bombarded with small spherical media named shot which brings local yield in tension on the steel surface. Moreover, the compressed grains below the surface tend to produce a hemisphere highly stressed location in compression. A uniform layer of residual compressive stress is then developed through overlapping dimples. The test shot peening was conducted by means of an injector type system using steel shot ball under the condition shown in Table 2. The coverage within the range of 200% and 400% was chosen in the present study. The surface condition after shot peening was evaluated in terms of micro-hardness measured by a hardness tester and in-depth compressive residual stress measured by an X-ray residual stress analyser (Pulstec Industrial Co., Ltd, Hamamatsu, Japan). In the latter examination, a series of nine repeated measurements were conducted without removing the specimen between successive measurements. During the measurement, the specimen was firstly positioned with integrated LED (Liquid Crystal Display) marker and CCD (Charge Coupled Device) camera (Hitachi, Ltd., Tokyo, Japan) for diffracted X-ray beams. Afterwards, the measuring data of X-ray diffraction are processed and the residual stresses are calculated automatically from the complete Deby ring information.

Fatigue tests were performed under constant amplitude sinusoidal stress cycles with the frequency of 8 Hz using a universal fatigue testing machine of 50 kN capacity. All test specimens were loaded in tension at room temperature in atmosphere environment. Constant stress ratio of 0.1 was kept for all tests. The fatigue life were determined as the number of cycles to failure and the maximum stress amplitude under which the specimen endured 10^7^ cycles respectively. Automatic data acquisition in the loading cycles was defined in the software controlling. The actual measured cross-sectional dimensions were used for the calculation of nominal stress of testing specimens.

## 3. Experimental TestResults

### 3.1. Fracture Surface Observation

**Figure 2:**
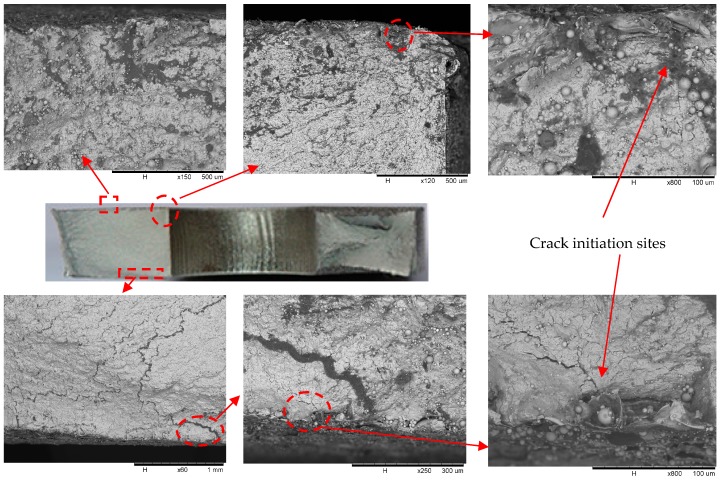
Typical fracture surface for test specimen (Δσ = 210 MPa).

All the test shot-peened open-hole steel plates are failed as the fatigue fracture took place at the edge of the open-hole. The fracture surfaces of test specimens were examined by means of scanning electron microscope (SEM) (Hitachi, Ltd., Tokyo, Japan). A typical observation at the stress range of 210 MPa is shown in Figure 2. As the fracture surface is magnified approximately 150~800 times, notable multiple crack initiations can be observed in the vicinity of the plate surface as well as the edge of the open-hole. Regarding the surface crack, the fatigue crack initiation size can be estimated in the range of 150~230 μm and 75~140 μm for shot-peened specimens with 200% and 400% coverage, respectively. It is the result of interest that the fatigue crack initiated and propagated not only from shot peening-induced roughening surface but also from the subsurface layers. As a result, the fatigue growth striations are not salient to the naked eye as reported for the structural component without shot peening [27]. Indeed, the fatigue cracks are prone to originate from the notches in the roughness profile but those are mitigated to some extent due to high compressive residual stress produced by shot peening. On the other hand, multiple cracks are propagated significantly at the edge of the open-hole region. At this point, high stress concentration can be expected and tensile fracture partially occurred as evidenced by some apparent necking. The subsurface layers are also subject to crack surface work hardening and compressive residual stresses induced by shot peening.

### 3.2. Surface Hardening Behaviour

**Figure 3:**
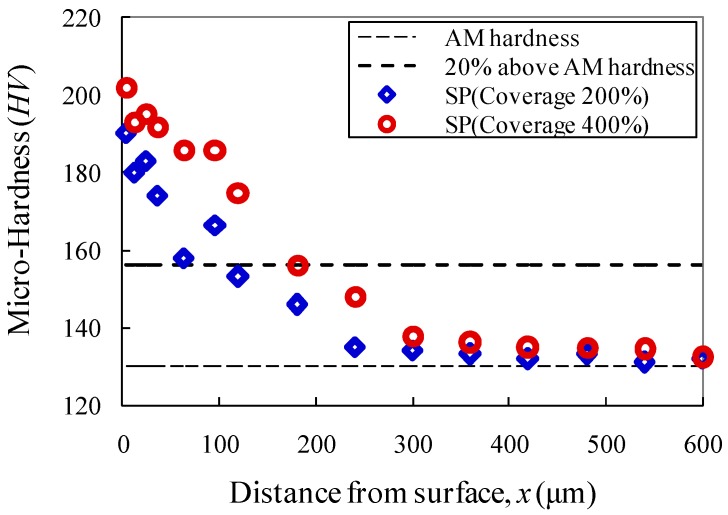
In-depth micro-hardness distribution of shot-peened specimens.

Typical variation of in-depth micro-hardness from the shot-peened surface is shown in Figure 3. The values of in-depth micro-hardness of shot-peened specimens were measured as it gradually reduced close to that of as-machined specimens. It can be seen that the scatters of measured values become less obvious as the micro-hardness ranges below 120% that of as-machined specimens. Moreover, the measured micro-hardness is decreased as close to that of as-machined specimen in the depth of approximately 600 μm. The beneficial effect of the coverage of shot peening on the distribution of micro-hardness can also be identified from the observation that the maximum micro-hardness is increased by nearly 10–17% as the coverage is increased from 200% to 400%. This improvement can be expected due to more condensed surface layers after the significant plastic deformation produced by stronger shot peening action.

**Figure 4:**
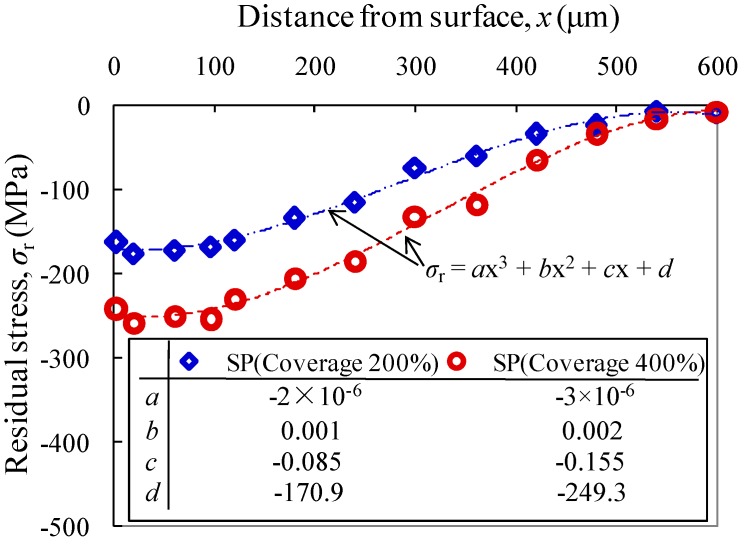
In-depth compressive residual stress distribution of shot-peened specimens.

Typical variation of in-depth compressive residual stress from the shot-peened surface is shown in Figure 4. The distribution of compressive residual stress ($\sigma_{r}$) can be expressed from treated surface (distance: *x*) using third-order polynomial regressions as:

(i) For shot peening with coverage of 200%:
  (1) $$\sigma_{r}=-2\times 10^{-6}x^{3}+0.001x^{2}-0.085x-170.9$$
(ii) For shot peening with coverage of 400%:
  (2) $$\sigma_{r}=-3\times 10^{-6}x^{3}+0.002x^{2}-0.155x-249.3$$

The resultant correlation coefficients are 0.994 and 0.993 for Equations (1) and (2), respectively. This indicates the effectiveness in the interpretation of damage progress using the expression in the form of fourth-order polynomial regression which is also in agreement with the findings reported by Sakamoto et al. [7]. The comparison of both expressions also shows that the distribution of compressive residual stress is varied with obvious gap as *x* is ranging between 0 and 350 μm, while its value is close to as-machined counterpart as *x* is approaching 350 μm.

### 3.3. S–N Relation and Analysis

Fatigue life results are normally expressed in terms of the relations between the stress range, Δσ, and the number of cycles to failure, *N*, as:

(3) $$N=C{\left(\Delta \sigma \right)}^{-m}$$

where, exponent, *m*, is the slope of the *S*–*N* relation; *C* is the material constant related parameter [3]. Taking logarithm on the both sides of Equation (3), the following equation can be written as:

(4) Log(N)=Log(C)−mLog(Δσ)

**Figure 5:**
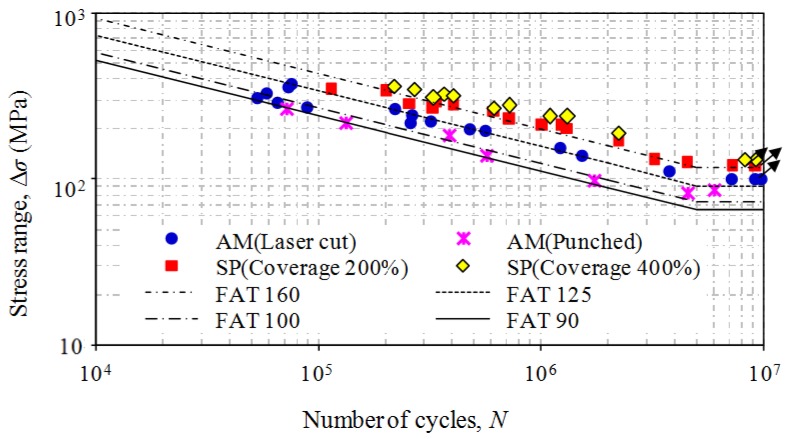
Comparison of *S*–*N* relations of shot-peened and bare steel open-hole details (AM: as-machined; SP: shot-peened).

Fatigue test data are summarized and plotted on a standard *S*–*N* diagram in Figure 5. Each data point was assigned the appropriate fatigue category, i.e., FAT 90, FAT 100, FAT 125, FAT 160, which represents the fatigue strength at 2 million cycles as codified in the EN 1993-1-9 [28]. The arrow denotes that the fatigue failure had not taken place when the tests were terminated at nearly 10^7^ cycles. The test data is fitted by the method of least squares in which the life results within fatigue limit is not taken into account in fitting the parameters. Using free regression analysis, the corresponding *S*–*N* relations for test specimens can be expressed as:

(i) For as-machined specimens:
  (5) Log(N)=14.71−3.958Log(Δσ)
(ii) For shot-peened specimens with 200% coverage:
  (6) Log(N)=15.46−4.051Log(Δσ)
(iii) For shot-peened specimens with 400% coverage:
  (7) Log(N)=14.85−3.705Log(Δσ)

The comparison of test data with assigned fatigue detail categories shows that the as-machined open-hole specimens can be classified to FAT 90, which agrees with the codified suggestion for drilled holes [28]. In contrast, the codified FAT classes are increased to125 and 160 for shot-peened specimens with 200% coverage and 400% coverage respectively. Such an increase amounts to 38% and 77% respectivel of the fatigue strength of bare open-hole steel plates

## 4. Discussion

The fatigue behaviour of shot-peened open-hole steel plates is significantly affected by surface hardening conditions induced by shot peening. The improvement of micro-hardness is varied with respect to the strength of steel materials. The maximum improvement of 20% against core hardness was reported by Hassani-Gangaraj et al. [6] when the high-strength low-alloy steel (878 MPa yield stress and 1010 MPa ultimate strength) was studied. For the sake of comparison, 20% improvement value was also superimposed in the graph of Figure 3. It is shown that the increase of micro-hardness is slightly greater than that reported in [6], which can be due to much higher plastic deformation and strain accumulation of the test materials with lower yield strength. It is also indicated in [6] that the combined effect of nitriding together with shot peening is effective in the enhancement of surface micro-hardness, e.g., nearly twice the improvement in contrast to core hardness. As such, nitriding treatment can be suggested as a supplement to the further improvement of surface hardness.

As shown in Figure 4, the maximum measured compressive residual stress is increased by nearly 50% as the coverage is increased from 200% to 400%. In contrast, the improvement of compressive residual stress is much greater than that of micro-hardness. This observation is in agreement with previous reports [5,6] that the shot peening seems more effective in the improvement of compressive residual stress than micro-hardness of the surface. Regarding this, optimum improvement of the maximum compressive residual stress can be anticipated as the surface is treated with shot peening in the increase of compressive residual stress followed by nitriding in the increase of micro-hardness.

As shown in Figure 5, the codified FAT 90 can be used for as-machined open-hole specimens. However, it is noted that the methods in open-hole production are not specified in the EN 1993-1-9 [28]; rather, the codified FAT 90 is represented for the details “Hole drilled or reamed”. As a comparison in Figure 5, the data of punched-hole details as reported by Garcia et al. [27] is slightly lower in contrast to FAT 90; therefore, measures are needed if the codified class has to be satisfied. For this case, it seems that the shot peening is by far useful in further enhancement of fatigue life of test specimens. This is because the crack initiation of punched hole is closely tied to the amount of disturbance in the hole surface caused by the punching process [29]. Accordingly, if the dimensions of surface flaws induced by punching are small in contrast to the shot peening affected area, the fatigue life of shot-peened specimens would not be affected by these flaws, and also the beneficial effect on life improvement would be realized. It is evident that the fatigue strengths can be increased by 38% and 78% of shot-peened specimens with 200% and 400% coverage respectively in contrast to as-machined ones. This increase effect is very close to that strengthened by CFRP as reported in [18]; however, the implementation of the technique of shot peening seems readily in contrast to CFRP, which are influenced by the bond strength of laminates.

For engineering application, further evaluation of beneficial effect for fatigue life is required. The fracture mechanics theory-based relation between fatigue crack propagation rate, da/dN and stress intensity factor, Δ*K*, can be written [30] as:

(8) $$\frac{da}{dN}=C{\left(\Delta K\right)}^{m}$$

**Figure 6:**
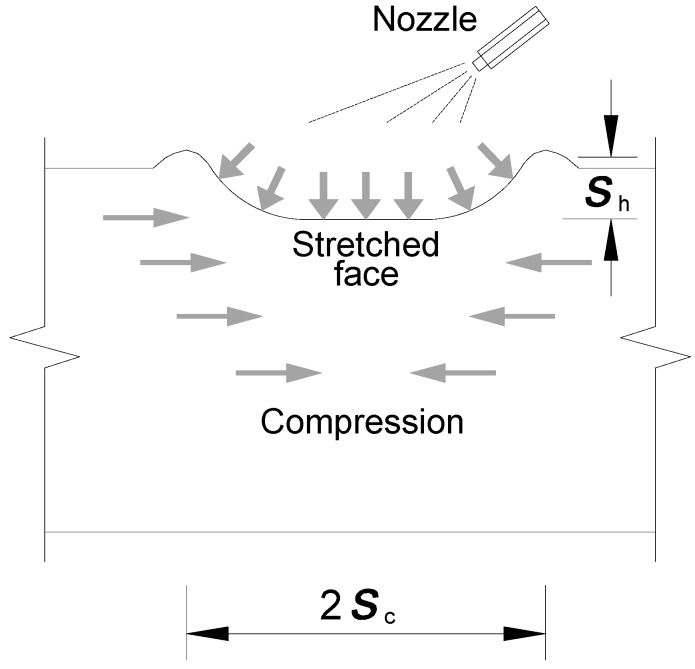
Height and width in a unit valley in roughness profile.

Taking integrating both sides of Equation (8) yields:

(9) $$N_{r}=\frac{1}{C}\int_{a_{i}}^{a_{f}}\frac{da}{\Delta K^{m}}$$

where $a_{i}$ and $a_{f}$ are the initial and final depths of the fatigue crack, respectively. *C* is the material constant-related parameter, which is taken as 2.18 × 10^−13^ [30]. *m* is the exponent related to the slope of the *S*–*N* relation which is taken as 3.0 in this study [31]. Δ*K* is the stress intensity factor range in unit of $\mathrm{MPa}\sqrt{m}$, which can be expressed using an asymptotic solution proposed by Liu and Mahadevan [17] as:

(10) $$\Delta K=1.122\Delta \sigma \sqrt{\pi \left(a+s_{h}\left[1-e^{\left[-\frac{a}{s_{h}}\left(K_{t}^{2}-1\right)\right]}\right]\right)}$$

where a is the crack length and $S_{h}$ is the notch depth. $K_{t}$ is the stress concentration factor as the product of the counterpart factors due to the roughening surface induced by shot peening ($K_{tr}$), the presence of open-hole ($K_{to}$) and the plate geometric condition ($K_{geo}$). $K_{tr}$ is the stress concentration factor related to the roughness profile which can be given as:

(11) $$K_{tr}=1+2.1\left(\frac{s_{h}}{2s_{c}}\right)$$

where $S_{c}$ is the notch half width in the roughness profile, as illustrated in Figure 6, which can be obtained from roughness measurements of peened surface using profilometer. The stress concentration factor ($K_{to}$) is defined as the ratio of the maximum tensile stress ($\sigma_{1,max}$) at the edge of the open-hole to the stress which is located far from the hole ($\sigma_{0}$). Regarding this, it is assumed that an open-hole plate herein is subjected to a uniform tension of magnitude of $\sigma_{0}$. The position of a point can be defined in a polar coordinate by the distance from the centre of the open-hole (i.e., radius *r*) and by the angle θ. The tangential stress in polar coordinates [2] can be obtained as:

(12) $$\sigma_{\theta }=\frac{\sigma_{0}}{2}\left(1+\frac{r_{\mathrm{bh}}^{2}}{r^{2}}\right)-\frac{\sigma_{0}}{2}\left(1+\frac{3r_{\mathrm{bh}}^{4}}{r^{4}}\right)\cos2\theta$$

Substituting $r=r_{bh}$ and θ = π/2 or 3π/2 into the above equation, the maximum tensile stress at the edge of the open-hole can also be calculated to be $\sigma_{1,max}=3\sigma_{0}$, i.e., stress concentration factor $K_{to}=3.0$.

(13) $$K_{to}=3$$

**Figure 7:**
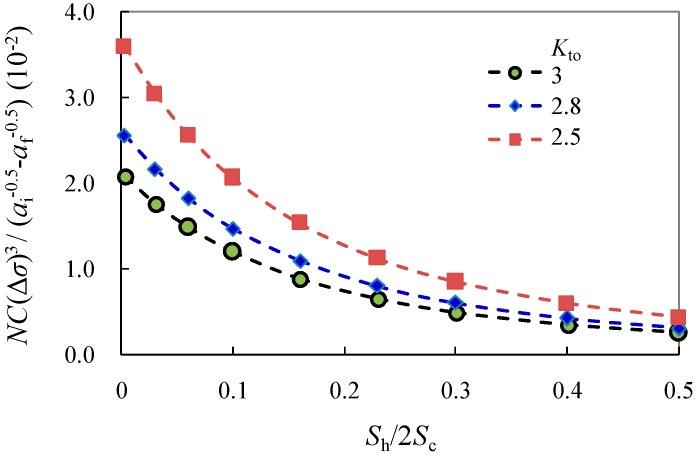
Relation between $NC{\left(\Delta \sigma \right)}^{3}/$$\left(a_{i}{}^{-0.5}-a_{f}{}^{-0.5}\right)$ and $S_{h}/2S_{c}$.

It is noted that the predicted fatigue life can be notably improved with the reduction of *K*_to_, as shown in Figure 7.

The correction factor for plate geometric condition ($K_{geo}$) allowing for finite thickness and width can be written as:

(14) $$K_{geo}=\left[1-0.025{\left(\frac{a}{2b_{0}}\right)}^{2}+0.06{\left(\frac{a}{2b_{0}}\right)}^{4}\right]\sqrt{\sin\left(\frac{\pi a}{4b_{0}}\right)}$$

where *b*_0_ is the half width of the plate.

Two conditions can be discussed as short crack and long crack [16,17]. In the former case, $a/s_{h}$ and $a/s_{h}\left(K_{t}^{2}-1\right)$ are approaching zero for a finite stress concentration factor, and then the exponential function can be simplified using the first-order Taylor series as:

(15) $$e^{\left[-\frac{a}{s_{h}}\left(K_{t}^{2}-1\right)\right]}=1-\frac{a}{s_{h}}\left(K_{t}^{2}-1\right)$$

Then, the Equation (10) can be rewritten as:

(16) $$\Delta K=1.122K_{t}\Delta \sigma \sqrt{\pi a}$$

In the latter case, $a/s_{h}$ and $a/s_{h}\left(K_{t}^{2}-1\right)$ are approaching infinity as $K_{t}$ is not equal to unity, and then Equation (15) is approaching zero; therefore, Equation (10) can be expressed as:

(17) $$\Delta K=1.122\Delta \sigma \sqrt{\pi (a+s_{h})}$$

From the discussion between Equations (15) and (17), it is worthy of note that only the former case of short crack is dependent on the stress concentration due to shot peening-induced surface roughening. As shown in Figure 7, the fatigue life is almost exponentially decreased with the increase of $S_{h}/2S_{c}$ related to notch profile. As such, this variation together with the effect due to open-hole details and geometric condition can be taken into account in the expression of stress concentration as:

(18) $$K_{t}=K_{\mathrm{tr}}K_{\mathrm{to}}K_{\mathrm{geo}}$$

Substituting Equations (16) and (18) into Equation (9), the fatigue life can be obtained as:

(19) $$N_{r}=\frac{1.42}{C{\left(K_{\mathrm{tr}}K_{\mathrm{to}}K_{\mathrm{geo}}\Delta \sigma \sqrt{\pi }\right)}^{3}}\left(\frac{1}{\sqrt{a_{i}^{}}}-\frac{1}{\sqrt{a_{f}^{}}}\right)$$

**Figure 8:**
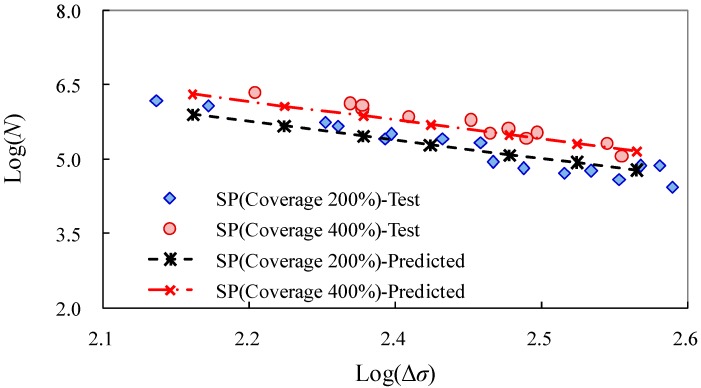
Comparison of test and predicted log(*N*)-log(Δσ) curves.

The dimension of the crack is estimated from fracture surface observation as initial depth $a_{i}$ in 150 μm and 75 μm for shot-peened specimens with 200% and 400% coverage respectively. The value of $a_{i}$ herein was obtained as the minimum crack size identified in the fracture surface observation as aforementioned in the Section 3.1. The final crack depth $a_{f}$ is equal to the plate thickness since the remaining life in crack propagation to the flange tip is negligible subsequent to that through the plate thickness. The fatigue life results for the test specimens with Δσ varying between 150 MPa and 320 MPa and shot peening coverage of 200% and 400% can then be predicted and plotted in the *S*–*N* relation. As shown in Figure 8, the predicted curves correlate well with fatigue test data, which indicates the ability of the aforementioned method in the evaluation of fatigue life of shot-peened open-hole steel plates.

## 5. Concluding Remarks

The fatigue behaviour of shot-peened open-hole steel plates has been presented in this paper. The characteristics of crack propagation at surface and subsurface layers were analyzed with the aid of scanning electron microscopy. The surface hardening effect produced by shot peening was studied in terms of the distributions of in-depth micro-hardness and compressive residual stress. Based on the test data, the fatigue result of shot-peened open-hole details has been compared with codified fatigue categories. Finally, a fracture mechanics theory-based analytical method in the prediction of test fatigue life has been proposed with due consideration of the stress concentrations related to the roughening surface, the presence of open-hole and the plate geometric condition. The following conclusions can be drawn:

The initiation of fatigue cracks are formed at the notches of roughening surface and subsurface layers due to shot-peened impact. Moreover, the fatigue crack propagation is mitigated to some extent due to high compressive residual stress produced by shot peening, which is amplified with the increase of the coverage of shot peening. It is shown that these effects are interacted with local stress concentration when the fatigue life of shot-peened open-hole details is evaluated.

The beneficial effect of surface hardening can be identified from shot-peened open-hole specimens when compared against as-machined counterparts. By contrast, it is shown that when the shot peening coverage is increased from 200% to 400%, the increase of compressive residual stress (nearly 50%) is much greater than micro-hardness (10–17%).

The comparison of test results revealed that the fatigue detail category of as-machined open-hole specimens (classified as FAT 90 in accordance with EN 1993-1-9) can be significantly improved to FAT 125 and FAT 160 for these with 200% and 400% shot peening coverage, respectively.

The predicted fatigue life results correlate well with test data which justifies the proposed method in the evaluation of fatigue life of open-hole steel plates under the condition of shot peening. Notwithstanding this, more experimental work is still needed to provide further improvement of the proposed method stated herein. Meanwhile, the evaluation of the shot peening effect on the fatigue crack growth of open-hole steel plates is another issue deserving future investigation.

## Figures and Tables

**Table 1:** Chemical composition and mechanical properties of steel at room temperature.

Steel Grade	Chemical Composition (%)					Mechanical Properties		
	C	Si	M_n_	P	S	σ_y_ (MPa)	*E*_s_ (MPa)	σ_u_ (Mpa)
Q345B	0.17	0.25	1.15	0.015	0.014	388	2.1 × 10^5^	553
S460M [22]	0.12	0.45	1.49	0.012	0.001	484	2.05 × 10^5^	594

**Table 2:** Shot peening conditions.

Shot ball			Shot Velocity	Shot Impact Angle	Distance	Pressure	Coverage
Material	Hardness	Diameter					
Steel	HRC 48–52	0.6 mm	150 mm	45	150 mm	5 kg/cm^2^	200% 400%
